# The influence of cereboost on mood, cognitive function, and simulated driving in professional race car drivers

**DOI:** 10.3389/fspor.2025.1658468

**Published:** 2025-09-09

**Authors:** David P. Ferguson, Pascale Franca-Berthon, Claire Williams, Romain Le Cozannet

**Affiliations:** ^1^ Department of Kinesiology, Michigan State University, East Lansing, MI, United States; ^2^ Givaudan France Naturals, Avignon, France; ^3^ School of Psychology & Clinical Language Sciences, University of Reading, Reading, England

**Keywords:** driver science, cognitive function, American Ginseng, motorsports, supplements

## Abstract

**Introduction:**

Race car driving is a physically and cognitively demanding sport requiring rapid decision-making under extreme conditions. While physical training and hydration strategies have been explored, few studies have investigated nutritional interventions to enhance cognitive or driving performance. This study examined the effects of Cereboost, an American Ginseng extract, on mood, cognitive function, and simulated driving performance in professional race car drivers.

**Methods:**

Fifty-eight licensed drivers completed a four-phase, within-subjects protocol (baseline, acute, chronic, and acute-on-chronic) involving 200 mg/day Cereboost supplementation. Assessments included validated mood questionnaires, cognitive testing via the Senaptec Sensory Station (spatial memory and split attention), and 30-minute sessions in a professional-grade racing simulator. Statistical analyses included ANOVA with Holm-Bonferroni corrections.

**Results:**

Cereboost had no statistically significant effects on mood or cognitive function after correction for multiple comparisons. However, acute-on-chronic supplementation significantly improved simulated driving performance, with a 3-second reduction in lap time and faster throttle application in corners (adjusted *P* = 0.000003, Cohen's *d* = –1.274). Participants reported subjective improvements in mental acuity (97%) and driving performance (94%).

**Conclusion:**

While Cereboost did not significantly alter mood or cognitive test outcomes, sustained supplementation enhanced simulated driving performance in professional drivers. These findings suggest potential benefits of nootropic supplementation for motorsport performance, warranting further investigation in on-track settings.

## Introduction

Automobile racing is a demanding sport where drivers are exposed to a variety of physical and cognitive stressors that result in a physiological response of burning 2,000 calories, sustaining heart rates of 60 to 70% maximum, increasing core body temperatures to 39 ℃, and losing 3 Kg of sweat during a three hour race (1–10). While the physical work to pilot the vehicle is substantial (11), Schwaberger proposed in 1987 that the emotional and cognitive stress placed on drivers has a significant influence on performance (12). Indeed, drivers are required to make split second decisions and if the incorrect decision is made the consequences could be impair driving performance and in severe cases lead to an crash (7, 10).

Despite the popularity of motor sports there have been extremely limited investigations into factors that could influence cognitive performance in race car drivers. The few examples have demonstrated that drivers benefit from cognitive training (13) and that the structural and functional aspects of a race car driver's brain are different from the general population (14, 15). To date there have been no studies focused on nutritional supplements that could improve cognitive function in race car drivers.

Cereboost (American Ginseng, manufactured by Givaudan) has adaptogenic properties, potentially offering several benefits for sports performance. American Ginseng (*Panax quinquelfolius*) has anti-inflammatory and antioxidant properties that help mitigate inflammation caused by intense physical activity (16). Research indicates that Cereboost can enhance executive function by influencing attention, recovery and reducing mental fatigue (16). The literature has demonstrated that cognitive capacity, and executive function can influence driving performance with errors occurring when there is a decline in executive function (17, 18). The influence of executive function on driving coupled with the fact that Cereboost is a natural supplement and not banned by any racing sanctioning body suggests that Cereboost could improve performance and safety of race car drivers. Therefore, we hypothesized that Cereboost would improve mood, cognitive function, and simulated driving performance.

## Methods

### Experimental design

The objective of the present investigation was to determine the effect of acute and chronic supplementation of Cereboost on cognitive and driving performance in race car drivers. The study was approved by the Michigan State University Institutional Review Board and conformed to the guidelines established by the Declaration of Helsinki and its later amendments. Prior to participant enrollment all participants reviewed and signed a university approved written informed consent document.

Professional race car drivers that were over 18 years old and currently competing in a professional series were recruited. Participants were recruited from Indianapolis, IN, USA, which has a high population of race car drivers and where data collection occurred. Participants traveled to the PitFit Training facility in Indianapolis multiple times to assess the influence of Cereboost on their mood, cognitive function, and simulator driving performance (detailed below). Throughout the study protocol participants had their food intake monitored using a cell phone application that calculated the calories, macronutrient, and micronutrient content of their diets. The data was used to determine if there were any potential nutritional influences (caffeine, malnourishment, etc.) on cognitive function.

The first PitFit visit prioritized participant acclimation where participants completed the assessments to limit any effect of learning on the outcome variables. Twenty-four hours later participants arrived in the morning and completed the tests as a “baseline measure”. The participant then consumed 200 mg of Cereboost [current dosage utilized in the literature to elicit improvements in mood at cognitive function—(16)] and returned to the facility two hours later. The participant then repeated the measurements for the “acute phase of testing”. Participants then consumed 200 mg of Cereboost once a day for two weeks. At the end of the two weeks participants returned to the PitFit facility and performed the measurements in the morning (Chronic measurement). The participants then consumed 200 mg of Cereboost and returned two hours later to repeat the measurements (Acute on Chronic measurement). Participants were compensated $250 for participation.

### Participants

A total of 129 licensed race car drivers were contacted about the study with seventy-eight people expressing interest in participating. Fifty-eight participants completed the acute testing with forty-two completing the chronic phase of testing. Ten percent of participants were female (representative of the race car driver population), and the average age of all participants was 26.4 ± 8.6 years.

### Mood questionnaires

Participants completed a series of validated mood questionnaires to determine the influence of Cereboost on their mood (16). The Calmness Likert Scale is a 9-point scale designed to measure an individual's current level of calmness. Participants were asked to rate their feelings in the present moment, ranging from “not at all calm” to “extremely calm”. The scale is used to assess the immediate emotional state of calmness.

The Mental Fatigue Likert Scale is a 9-point scale designed to measure an individual's current level of mental fatigue. Participants were asked to rate their feelings of mental fatigue in the present moment, ranging from “not at all mentally fatigued” to “extremely mentally fatigued”. The scale is used to assess the immediate cognitive state of fatigue.

The Physical Fatigue Likert Scale is a 9-point scale designed to measure an individual's current level of physical fatigue. Participants were asked to rate their feelings of physical fatigue in the present moment, ranging from “not at all physically fatigued” to “extremely physically fatigued”. The scale is used to assess the immediate physical state of fatigue.

The Bond-Lader Visual Analogue Mood Scales (VAMS) are designed to assess an individual's current mood state. This questionnaire consists of 16 94-mm lines, each anchored by antonyms (e.g., alert–drowsy, calm–excited). Participants mark their subjective state on these lines, and the distance from the negative antonym is measured in millimeters. The scores are then averaged to derive three primary mood factors: alertness, calmness, and contentedness. These scales are highly reliable and valid, originally developed to evaluate the mood effects of anxiolytics and have been widely used in pharmacological and psychopharmacological research (16).

The participants then completed a 94 mm scale that assesses stress and anxiety.

### Senaptec sensory station

Otto Lappi is a pioneer in understanding the cognitive capacity of race car drivers and has developed protocols to assess drivers (14, 15). These protocols have been utilized in various race car driver training studies with the Senaptec Sensory Station (Beaverton, OR, USA) (13). PitFit training is one facility that utilizes the Senaptec Sensory Station as a reliable and robust tool to assess cognitive function in race car drivers. Therefore, the following two tests were used from the Senaptec Sensory Station to evaluate the influence of Cereboost on cognitive function. The participants completed two rounds of testing at each visit. For the “Spatial Memory Test 2” each trial was recorded separately while the “Split Attention Test 1” had the results averaged for each condition (baseline, acute, chronic, and acute on chronic).

The “Spatial Memory Test 2” on the Senaptec Sensory Station measures an individual's ability to remember and recall the location of visual stimuli. This test assesses spatial awareness and memory by presenting a series of visual targets that the user must remember and then accurately identify after a brief delay. This test is particularly useful for evaluating and training cognitive functions related to spatial memory, which are crucial for activities that require precise spatial awareness and navigation (race car driving). A higher score on the spatial memory test indicates improved memory and recall.

The “Split Attention Test 1” on the Senaptec Sensory Station measures an individual's ability to manage and respond to multiple tasks simultaneously. This test combines a central cognitive task with a peripheral motor task. Participants must respond to a constantly changing task at the center of the screen (a letter) while also reacting to peripheral targets (colored dots) appearing around the screen. This test is designed to evaluate and train the ability to divide attention effectively, which is crucial for activities that require multitasking and quick decision-making (race car driving).

The outcome measures of the split Attention test are:

1. **Total**: The total number of targets presented during the test. A higher number of targets indicates the participant completed the task at a quicker speed.
2. **Go Hit**: The number of correct responses to “Go” targets, indicating successful identification and reaction.
3. **No Go Hit**: The number of correct inhibitions to “No Go” targets, showing the ability to withhold a response when necessary.
4. **Late**: The number of responses that were too slow or delayed, indicating a lapse in reaction time.
5. **Overall Accuracy**: The percentage of correct responses out of the total number of targets, reflecting the user's overall performance.
6. **Precision**: The accuracy of responses in terms of hitting the correct targets without false positives.
7. **Speed**: The average reaction time to the targets, measuring how quickly the user can respond.

### Racing simulator

Participants drove for 30 min on a racing simulator in a Ferrari 488 GT3 Evo at the Road America track, which has been previously demonstrated to be a valid tool to assess race car driving performance (1). The car setup and track conditions were identical for all participants and phases of Cereboost supplementation. Lap time, driving errors, and number of laps completed were recorded. Additionally, the full race car telemetry system was downloaded to provide insight into driving performance. Specifically, we evaluated the throttle, brake, and steering responses to determine if Cereboost use influenced driving behavior.

### Satisfaction survey

At the end of the study all participants were asked to rate how much they agree with nine statements regarding Cereboost to assess their satisfaction with the product. The reason to include the survey was that the routines of race car drivers are unique to other sports and understanding if supplement use can be used in racing is understudied. Responses were rounded to the nearest percent.

### Statistics

All data was analyzed in JMP Pro v16.0 (Sass, Cary, NC). Normality of the data was confirmed and then an Analysis of Variance (ANOVA) was performed comparing condition (baseline, acute, chronic, and acute on chronic) to the variables defined above. Furthermore, data is presented as raw values and percentage change from baseline. An alpha level of 0.05 was set *a priori* and if significant (*P* ≤ 0.05) a Tukey's HSD *post hoc* test was run. We then performed a Holm-Bonferroni correction to control for familywise error rate. All values are presented as mean ± standard error.

## Results

### Mood questionnaire

Table 1 indicates that for all tests the participants were calm and not mentally or physically fatigued. There was no influence of Cereboost conditions on calmness or fatigue. Table 2 indicates that the participants were alert, calm and slightly discontented during the testing procedures. There was no influence of Cereboost conditions on alertness, calmness, or contentedness. Table 3 indicates that the participants were not stressed or anxious during the testing procedures and that there was no influence of Cereboost on stress or anxiety.

**Table 1:** 9-point Likert scales.

Condition	Calmness	Mental fatigue	Physical fatigue
Baseline	6.9 ± 0.2	3.2 ± 0.5	2.9 ± 0.6
Acute	7.0 ± 0.3 (+1.4)	3.1 ± 0.5 (−3.1)	2.9 ± 0.6 (+0.0)
Chronic	6.6 ± 0.6 (−4.3)	2.8 ± 0.6 (−12.5)	2.4 ± 0.5 (−17.2)
Acute on Chronic	7.3 ± 0.3 (+5.8)	2.4 ± 0.5 (−25.0)	2.4 ± 0.5 (−17.2)

The closer the values are to 9 indicate the participants were calmer or more fatigued. There was no differences for calmness (*P* = 0.61), mental fatigue (*P* = 0.78), or physical fatigue (*P* = 0.85) between conditions. Data is presented as raw values with the percentage change from baseline in parentheses.

**Table 2:** Bond-lader visual analogue mood scales.

Condition	Alertness	Calmness	Contentedness
Baseline	68.1 ± 3.4	59.8 ± 4.7	24.5 ± 4.1
Acute	74.2 ± 2.7 (+8.9)	59.9 ± 3.4 (+0.1)	24.1 ± 4.9 (−1.6)
Chronic	67.5 ± 3.8 (−0.8)	61.3 ± 6.1 (+2.5)	21.8 ± 4.7 (−11.0)
Acute on Chronic	73.9 ± 4.5 (+8.52)	60.9 ± 6.0 (+1.8)	19.4 ± 3.8 (−20.8)

The scale is out of 94, with values closer to 94 indicated a state of more alertness, calmness, contentedness. There was no effect of condition on alertness (*P* = 0.40), calmness (*P* = 0.99), or contentedness (*P* = 0.79). Data is presented as raw values with the percentage change from baseline in parentheses.

**Table 3:** Mood scale of stress and anxiety.

Condition	Stress	Anxious
Baseline	23.9 ± 5.4	23.1 ± 5.5
Acute	19.5 ± 5.1 (−18.4)	21.6 ± 5.2 (−6.4)
Chronic	22.7 ± 6.4 (−5.0)	25.0 ± 7.4 (+8.2)
Acute on Chronic	21.1 ± 6.3 (−11.7)	24.3 ± 6.0 (+5.1)

The scale is out of 94, with values closer to 94 indicated a state of more stress or anxious. There was no effect of condition on stress (*P* = 0.96) or anxiety (*P* = 0.97). Data is presented as raw values with the percentage change from baseline in parentheses.

To control for the familywise error rate across multiple mood and fatigue survey comparisons, Holm-Bonferroni corrections were applied to all *post hoc P*-values. Across all measures—including calmness, mental fatigue, physical fatigue, alertness, contentedness, and stress/anxiety—none of the comparisons were statistically significant after correction.

### Senaptec sensory station

The results from the Senaptec Sensory Station are displayed in Tables 4, 5. There was no influence of Cereboost on spatial memory or split attention. However, there was an effect (*P* = 0.05) of Cereboost for the “late” variable on the split attention test (Table 5), indicating that chronic and acute on chronic conditions reduced reaction time by 86.6 and 80.0%, respectively, compared to baseline.

**Table 4:** Spatial memory test 2.

Condition	Trial 1	Trial 2
Baseline	17,461.8 ± 358.7	17,126.4 ± 435.3
Acute	18,131.7 ± 358.7 (+3.8)	17,825.6 ± 435.4 (+4.0)
Chronic	18,172.0 ± 404.6 (+4.0)	18,232.5 ± 491.1 (+6.4)
Acute on Chronic	18,539.3 ± 447.4 (+6.1)	18,431.3 ± 542.9 (+7.6)

There was no influence of Cereboost condition on spatial memory during trial 1 (*P* = 0.28) or trial 2 (*P* = 0.70). Data is presented as raw values with the percentage change from baseline in parentheses.

**Table 5:** Split attention test 1.

Condition	Total	Go Hit	No Go Hit	Late	Accuracy (%)	Precision (mm)	Speed (ms)
*Raw data*							
Baseline	173.7 ± 5.2	197.0 ± 1.2	1.8 ± 0.5	1.5 ± 0.4	89.8 ± 1.5	944.8 ± 7.5	1,589.8 ± 40.2
Acute	171.6 ± 4.8 (−1.2)	197.1 ± 1.1 (+0.0)	1.8 ± 0.4 (+0.0)	1.4 ± 0.4 (−6.6)	91.3 ± 1.4 (+1.6)	947.7 ± 7.0 (+0.3)	1,522.4 ± 37.2 (−4.2)
Chronic	179.6 ± 5.4 (+3.4)	194.0 ± 1.2 (−1.5)	1.8 ± 0.5 (+0.0)	0.2 ± 0.4 (−86.6)	93.5 ± 1.6 (+4.1)	951.0 ± 7.9 (+0.6)	1,538.2 ± 42.0 (−3.2)
Acute on Chronic	184.7 ± 6.0 (+6.3)	196.3 ± 1.4 (−0.36)	0.9 ± 0.5 (−50.0)	0.3 ± 0.5 (−80.0)	94.4 ± 1.8 (+5.1)	941.8 ± 8.7 (−0.3)	1,462.6 ± 46.5 (−8.0)

There was no effect of Cereboost condition on total (*P* = 0.33), go hit (*P* = 0.25), no go hit (*P* = 0.52), late (*P* = 0.05), accuracy (*P* = 0.18), precision (*P* = 0.87), or speed (*P* = 0.24). Data is presented as raw values with the percentage change from baseline in parentheses.

To account for multiple comparisons across cognitive performance metrics, Holm-Bonferroni corrections were applied to the *P*-values from the Senaptec Sensory Station Split Attention Test. Although the “Late” response variable initially approached significance (raw *P* = 0.05), the adjusted *P*-value (Holm-Bonferroni *P* = 0.35) did not meet the threshold for statistical significance. All other outcome measures—including Total responses, Go Hit, No Go Hit, Accuracy, Precision, and Speed—also failed to reach significance after correction (all adjusted *P* ≥ 0.87). These results suggest that Cereboost supplementation did not produce statistically reliable improvements in multitasking or attentional control as measured by this cognitive test.

### Racing simulator

Professional drivers in an actual Ferrari 488 GT3 Evo racing on the Road America track will have racing laps lasting 130 s with qualifying laps lasting 124 s (based on skill and track conditions) (1). The participants in the present investigation completed the simulator laps in a similar time confirming they possessed skills of professional race car drivers. There was no influence of Cereboost on fastest lap time, number of driving errors, or number of laps completed (Table 6). However, the acute on chronic condition elicited a three second faster lap time than the other conditions, with reduced driving errors. After applying the Holm-Bonferroni correction to control for familywise error rate, the acute-on-chronic vs. baseline [Cohen's d = −1.274, 95% CI (−1.781, −0.767), adjusted *P* = 0.000003] comparisons became statistically significant. These results indicate large and robust effects of sustained Cereboost use on simulated driving performance. In contrast, the acute vs. baseline comparison did not reach significance [Cohen's d = −0.204, 95% CI (−0.634, 0.226), adjusted *P* = 1.000].

**Table 6:** Simulator driving performance.

Condition	Fastest lap time (seconds)	Driving errors (*n*)	Laps completed (*n*)
Baseline	130.0 ± 1.6	8.1 ± 1.9	12.1 ± 0.4
Acute	129.1 ± 1.6 (−0.69)	6.7 ± 1.9 (+45.7)	12.6 ± 0.4 (+4.1)
Chronic	129.7 ± 1.7 (−0.23)	11.8 ± 1.9 (−17.3)	11.8 ± 0.4 (−2.5)
Acute on Chronic	126.9 ± 1.9 (−2.4)*	8.6 ± 2.3 (+6.2)	12.8 ± 0.5 (+5.8)

There was no influence of Cereboost condition on fastest lap time (*P* = 0.62), driving errors (*P* = 0.29), or laps completed (*P* = 0.38). *Indicates significance was achieved with a Holm-Bonferroni correction. Data is presented as raw values with the percentage change from baseline in parentheses.

**Figure 1:**
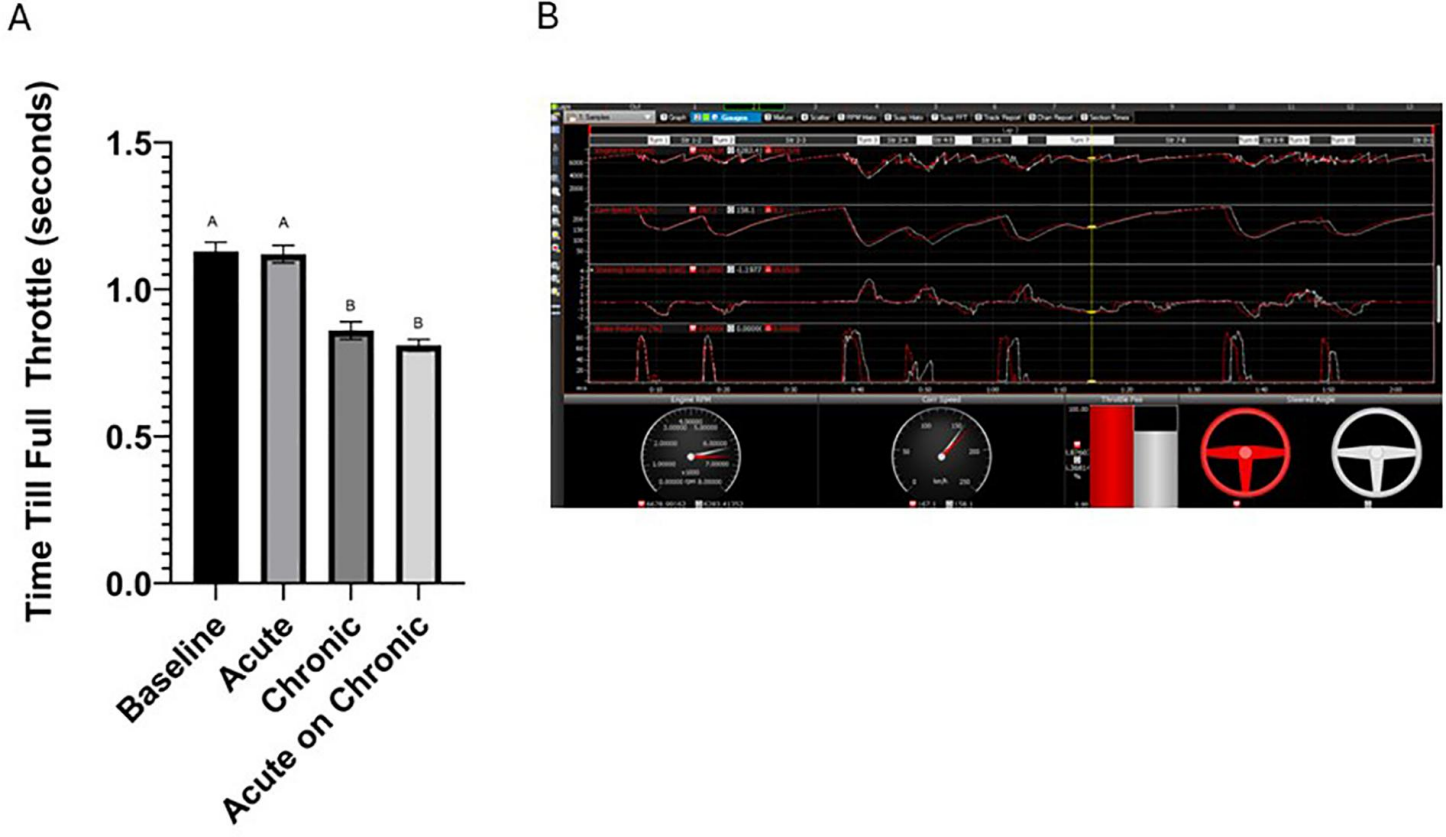
**(A)** Time to full throttle in the corners of the Road America Track for each condition. Differing letters indicate statistical significance (*P* = 0.03). **(B)** A representative telemetry tracing of one participant on the Road America Track. The white colored lines represent the acute condition, and the red colored lines represent the acute on chronic condition. The image highlights turn seven where the acute on chronic condition resulted in a faster time to 100% throttle.

When examining the telemetry data from the simulated driving sessions, it was determined that participants during the chronic and acute on chronic conditions reached 100% throttle in the corners faster (Figure 1A, *P* = 0.03) than participants in the baseline and acute conditions. The Road America racecourse contains ten turns that require drivers to slow down before entering the turn. To achieve the fastest lap time, drivers must travel through the corners as quickly as possible and the ability to generate 100% throttle in the corners will elicit the fastest lap times. A representative telemetry tracing is presented in Figure 1B, where the white colored lines represent the acute condition, and the red colored lines represent the acute on chronic condition for the same participant. The visual display indicates that the participant obtained 100% throttle faster in the acute on chronic condition which resulted in a faster speed through the corner and an overall faster lap time.

### Food tracking

All participants consumed similar calories, macro- and micronutrients during the experimental protocol. Participants did not consume any supplements that are marketed to enhance cognitive function. If participants consumed caffeine they did not deviate from their daily dosage throughout the study.

### Satisfaction survey

Table 7 depicts that participants found Cereboost easy to use, and they felt it improved their performance. Additionally, drivers would consider incorporating Cereboost into their training regimen.

**Table 7:** Participant satisfaction with cereboost.

Survey statements	Strongly agree	Agree	Neither agree nor disagree	Disagree	Strongly disagree
Cereboost is easy to use	97	3	0	0	0
Cereboost helped improved my mental performance	82	15	3	0	0
Cereboost was effective in improving my driving performance	49	45	6	0	0
Cereboost was effective in improving my focus	64	30	6	0	0
Cereboost was more effective than other products I have tried in the past	33	42	15	10	0
I perceived the effect of Cereboost faster than other products I have tried in the past	64	27	9	0	0
I am satisfied with Cereboost	79	15	6	0	0
I could quickly feel the effect of Cereboost	70	21	9	0	0
I would like to incorporate Cereboost in my training routine	40	51	9	0	0

## Discussion

Automobile racing is a physically and cognitively demanding sport where drivers must pilot vehicles at high rates of speed while being exposed to elevated ambient temperatures gravitational forces, and vibration (7, 19). The exposures placed on drivers during competition can impair performance and in extreme situations increase the risk of an on track incident (10). Unlike more traditional sports (football, basketball, and baseball), there are less than 40 peer reviewed publications on the physiological demands and evidence based therapeutic countermeasures that influence race car driver performance (7). The existing evidence indicates that physical training and nutrition practices that reduce physical fatigue while driving can optimize performance (1, 3, 7, 19–23).

Little empirical research has been conducted on strategies to prevent cognitive fatigue in racing car drivers. Cereboost, an extract derived from American Ginseng, has been clinically proven to offer several cognitive benefits, where studies have shown that it can enhance memory, attention, energy, and mood without the need for caffeine (16, 24). The active compounds in Cereboost are responsible for these effects and can start working within an hour of consumption (16). The present investigation hypothesized that Cereboost could improve mood, cognitive performance, and simulated race car driving in professional race car drivers.

The results demonstrated that acute and chronic supplementation with Cereboost had no effect on mood or cognitive performance (Senaptec Sensory Station). However, there was an initial effect (*P* = 0.05) where chronic and acute on chronic conditions resulted in improved reaction time on the Split Attention Test (Table 5), but that effect was lost when further evaluated with the Holm-Bonferroni correction.

Following chronic supplementation with Cereboost, participants were able to generate “full throttle” in the corners of the race track faster than the baseline or acute condition (Figure 1A). In road course racing (tracks with left and right hand turns), one of the keys to success is the ability of the race car to travel through the corners as fast as possible (25, 26). Thereby the sooner an individual can obtain “full throttle” (100% depression of the throttle pedal) in the corner the faster their lap time will be (26). Indeed, in the acute on chronic condition participants reached full throttle sooner which increased cornering speed, and decreased lap times by three seconds (Table 6). The performance improvement is crucial for the motorsport community as elite level IndyCar teams will spend $100,000 to gain 0.1 s at the Indianapolis 500 (27).

There is a potential that the driving performance observed on the simulator was a result of learning. Previous investigations on racing simulators demonstrate that in professional drivers that have experience with simulators and driven the actual track have a plateau in learning (14, 15). All drivers in the present investigation were familiar with simulators and had driven the Road America track on simulators and real life which limits the effect of learning in the present investigation. Thereby, chronic supplementation with Cereboost could be the primary factor responsible for performance enhancement seen in this study.

Drivers reported a subjective sense of improved mental acuity (97%) and driving capabilities (94%) following supplementation with Cereboost. This feeling of heightened performance is crucial in the context of racing, where the psychological aspect can significantly influence a driver's confidence and decision-making on the track (28). The drivers noted that they felt more alert and responsive, which aligns with the observed improvements in their results on the simulator trials. Such subjective experiences of enhanced performance are vital, as they can reinforce a driver's belief in their abilities, potentially leading to better outcomes during competition. This combination of subjective and objective outcomes is relevant for evaluating nutritional interventions aimed at athletes; it is essential that the benefits are not only scientifically validated but also perceived by the users themselves (28). The ability to feel improvements quickly (91%) and faster than other solutions (91%) can serve as a motivating factor for drivers, encouraging them to incorporate effective nutritional strategies like Cereboost into their training regimens, thereby optimizing both their mental and physical performance on the track.

### Limitations

While there was an improvement in driving performance it was surprising that Cereboost had no effect on the mood or cognitive measures despite the supplement demonstrating such effects previously (16). In certain outcome variables there were sizable percentage improvements from baseline, which did not reach statistical significance. In order to have a moderate effect size (Cohen's d = 0.5) 37 participants were required. Thus, the present investigation has a moderate effect size. In order to have a small effect size (Cohen's d = 0.2) 266 participants were required, which is not achievable in the Indianapolis area. Furthermore, it is for this reason that a placebo-controlled trial was not performed as there were not enough participants in the Indianapolis area to have a control and experimental condition. Another aspect of sample size is several participants withdrew from the study due to the travel demands of their racing schedule (they were no longer in the Indianapolis area).

The lack of significance could be because race car drivers respond to Cereboost differently the general population. The participants in the present investigation were a young highly cognitively functioning group. Race car drivers are known to have enhanced reaction time, response accuracy, and cognitive process compared to the general population (7, 14, 15, 27), thus it is possible Cereboost had a minimal influence on their mood which is already optimized. As drivers age, they could see greater benefits with Cereboost as compared to the population in the present investigation. Therefore, it is important for future studies to evaluate older drivers and drivers with varying levels of racing experience.

## Conclusions

Cereboost has previously demonstrated its ability to reduce mental fatigue and enhance attention and cognitive performance (16). In this study, Cereboost had a positive impact on driving performance, including increased time to full throttle and reduced lap times—both vital for the racing community. This research not only establishes a connection between mental performance and athletic performance but also highlights, for the first time, the beneficial effects of nootropics like Cereboost on athletic performance. The next step in this line of research is to perform follow-up on-track analysis to confirm the simulator results translated to actual racing.

## Data Availability

The raw data supporting the conclusions of this article will be made available by the authors, without undue reservation.
